# Acoustic-Emission-Based Multiscale Tensile Constitutive Model for Ultra-High-Performance Concrete Considering Steel-Fiber Parameters and Beam-Scale Validation

**DOI:** 10.3390/ma19112428

**Published:** 2026-06-05

**Authors:** Zhenyu Bao, Qing Wang, Jinlan Deng, Meng Zhang

**Affiliations:** 1 Hubei Key Laboratory of Disaster Prevention and Mitigation, Yichang 443002, China; 13487279192@163.com (Z.B.); shuyuemingzheng@outlook.com (J.D.); 18871769782@163.com (M.Z.); 2 College of Civil Engineering & Architecture, China Three Gorges University, Yichang 443002, China

**Keywords:** ultra-high-performance concrete, damage variable, acoustic emission, uniaxial tensile test, tensile constitutive relationship, multiscale analysis

## Abstract

Ultra-high-performance concrete (UHPC) has attracted extensive attention because of its superior mechanical performance and durability. However, many existing tensile constitutive models are still obtained mainly by fitting macroscopic stress–strain curves, and the coupling among tensile damage development, steel-fiber parameters, and structural-scale response has not been sufficiently clarified. In this work, an acoustic-emission-informed tensile damage model was established for UHPC. Direct tensile tests were carried out on UHPC specimens containing steel fibers with aspect ratios of 43, 65, and 100 and volume fractions ranging from 0.5% to 3.0%, while acoustic emission signals were collected during loading. The normalized cumulative AE count was adopted as a damage indicator, and its evolution with tensile strain was described using a Weibull-type function. A fiber factor combining fiber volume fraction and aspect ratio was further incorporated into the damage constitutive equation. The proposed relationship was checked against 14 independent tensile datasets reported in the literature. After correction, the mean relative error of the predicted model parameter was reduced to 2.6%, with a standard deviation of 4.1%, and the fitted stress–strain curves all achieved R^2^ values above 0.85. The constitutive model was then implemented in ABAQUS for the simulation of reinforced UHPC beams. By introducing a member-level reduction coefficient of μ = 0.84, the numerical load–deflection curve showed improved agreement with the experimental beam response. The coefficient is empirical and is applicable only to the beam configuration investigated here unless further validation is performed. Overall, the proposed model provides a damage-based link among AE monitoring, steel-fiber reinforcement parameters, and member-scale numerical analysis.

## 1. Introduction

Ultra-high-performance concrete is a dense fiber-reinforced cementitious composite that has been increasingly used in structural and repair engineering. Owing to its high compressive strength, improved durability, and enhanced tensile resistance provided by steel fibers, UHPC has been widely considered for bridge structures, building components, and repair applications [1,2]. Unlike ordinary concrete, UHPC with sufficient fiber reinforcement may exhibit tensile strain-hardening and multiple-cracking behavior. Therefore, its tensile stress–strain response is quite different from that of normal concrete and cannot be represented adequately by conventional concrete constitutive laws [3,4,5,6,7]. A reliable uniaxial tensile constitutive relationship is essential for nonlinear finite element analysis and for the design and performance evaluation of UHPC structural members.

Previous tensile constitutive models for UHPC have generally been established by fitting experimental stress–strain curves. Such models are useful for reproducing test results, but their parameters are often empirical and are not always connected to the physical damage process occurring during loading. Piecewise tensile constitutive equations have been proposed by Shao et al. [8] and Hu et al. [9] based on uniaxial tensile test data. Other researchers defined damage variables according to stiffness degradation or secant modulus reduction and developed tensile damage constitutive models for UHPC or UHPFRC [10,11,12]. Ma et al. [13] further considered an exponential relationship between the UHPC damage factor and elastic–plastic strain and verified the model through finite element analysis. However, the influence of steel-fiber geometry and dosage is still commonly considered indirectly, and damage information obtained from acoustic emission monitoring has not been fully integrated into tensile constitutive modeling.

In this study, the term “multiscale” is used to describe three connected analytical levels rather than direct microstructural characterization of hydration products or pore structure. These levels include AE-inferred damage evolution during crack initiation and propagation, the reinforcing effect associated with steel-fiber volume fraction and aspect ratio, and beam-scale verification of the proposed tensile constitutive model. Existing tensile damage variables are commonly defined from macroscopic mechanical responses, including stiffness degradation and secant modulus reduction. Although such approaches can characterize the overall deterioration of tensile behavior, they do not directly reflect real-time microcracking activity during loading. Therefore, this study introduces an AE-informed damage variable to establish a closer connection between tensile damage evolution and constitutive modeling.

Recent studies have improved the understanding of UHPC tensile constitutive behavior, AE-based damage identification, and steel-fiber orientation effects. For instance, tensile constitutive studies have considered fiber volume fraction, fiber orientation, and post-cracking response, whereas AE-based studies have employed cumulative counts, energy, peak frequency, RA value, AF value, and b-value to distinguish damage stages and crack modes [7,14,15,16,17,18,19]. However, a unified tensile constitutive framework that converts AE monitoring information into a physically interpretable damage variable while also incorporating steel-fiber volume fraction and aspect ratio is still insufficiently developed. Le Hoang and Fehling investigated the influence of steel-fiber content and aspect ratio on the tensile and compressive behavior of ultra-high-performance concrete, and Yoo and Banthia summarized the broader effects of fiber characteristics on the mechanical performance of ultra-high-performance fiber-reinforced concrete [7,14,15,16,17,18,19]. Nevertheless, these studies mainly focused on mechanical responses, and the quantitative relationship among fiber parameters, AE-based damage evolution, and constitutive model parameters remains unclear. Hence, a tensile constitutive model that explicitly links AE-inferred damage evolution with steel-fiber parameters and can be further examined at the structural-member scale is still needed.

Acoustic emission has been widely applied to monitor damage development in cement-based materials. For ultra-high-performance fiber-reinforced concrete, previous AE studies have identified tensile damage stages and damage sources, including matrix cracking, fiber–matrix debonding, and fiber pullout [20,21]. However, only limited work has converted AE cumulative count information into a physically meaningful tensile damage variable and then incorporated it into a constitutive model considering steel-fiber parameters [22,23]. Chen et al. [3,24] and Wang et al. [25,26] investigated the internal damage mechanism and evolution process of UHPC using AE parameters such as source location, cumulative count, and energy. In the present study, the microscale damage process refers to crack initiation and propagation inferred from AE activity, rather than direct microscopic observations such as scanning electron microscopy of hydration products or pores.

The objective of this study is to establish a tensile constitutive model for ultra-high-performance concrete in which the damage variable is derived from AE monitoring and the model parameter is related to steel-fiber volume fraction and aspect ratio [27,28]. The novelty of this work lies in integrating AE-based damage evolution, fiber-parameter-dependent constitutive modeling, and beam-scale finite element validation within a single framework. To investigate the effect of steel-fiber parameters on the specimen-scale tensile constitutive response, uniaxial tensile tests were conducted on UHPC specimens with different fiber aspect ratios and volume fractions. The relationship between the steel-fiber factor and the constitutive model parameter was then obtained through regression analysis. Based on the AE-derived damage variable and the fiber factor, a tensile damage constitutive model for UHPC was proposed. Finally, the applicability of the proposed model was evaluated by simulating the flexural response of reinforced UHPC beams, and a member-scale reduction coefficient was introduced for beam-scale validation [29,30].

## 2. Experiments

The experimental program linked AE-based damage characterization, fiber-parameter calibration, and beam-scale verification. Detailed practices for UHPC preparation, tensile testing, AE acquisition, and beam loading followed established procedures reported in; only the parameters essential to the present model calibration are summarized below.

### 2.1. Test Scheme

#### 2.1.1. Materials and Mixture Proportion

Commercial silica fume, polycarboxylate superplasticizer, natural river sand, and copper-plated micro steel fibers were used. Two UHPC matrices were adopted: U1 for AE damage analysis and beam validation, and U2 for the fiber-parameter study. Because the material-characterization and UHPC mixture-design procedures are well documented in previous studies, this section retains only the key mixture information needed for reproducibility [31,32].

The principal properties of the admixture, sand, and steel fibers are consistent with UHPC systems reported in the literature. The mixture proportions are given as normalized mass ratios in Table 1 and Table 2.

The ratios in Table 1 and Table 2 are normalized by binder or cement mass, following the reporting conventions commonly used for UHPC mixtures.

#### 2.1.2. Experimental Design

For AE damage analysis, U1 specimens with steel fibers of aspect ratio 65 were prepared at volume fractions of 0.5%, 1.0%, and 1.5%.

**Figure 1:**
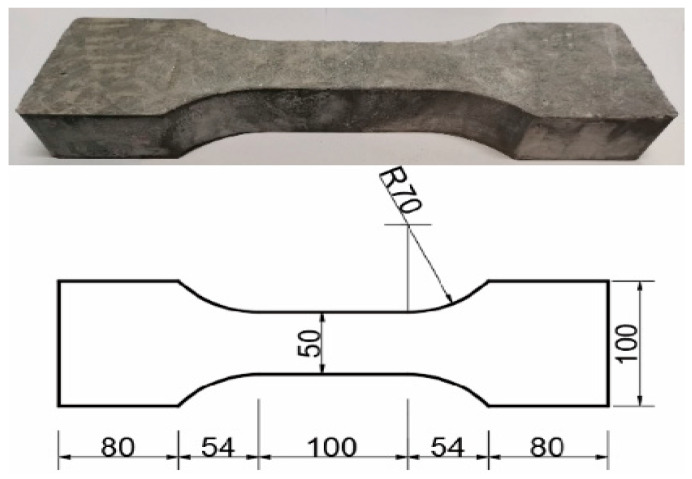
Dimensions of dog-bone-shaped specimen.

For fiber-parameter calibration, U2 specimens combined three fiber aspect ratios (43, 65, and 100) with six volume fractions (0.5–3.0%). Dog-bone specimens with a 50 mm × 50 mm gauge section and an overall length of 368 mm were adopted according to relevant direct-tension testing practice and previous UHPC studies, as shown in Figure 1.

Table 3 summarizes the specimen matrix; labels indicate matrix type, fiber aspect ratio, and fiber volume fraction.

**Figure 2:**
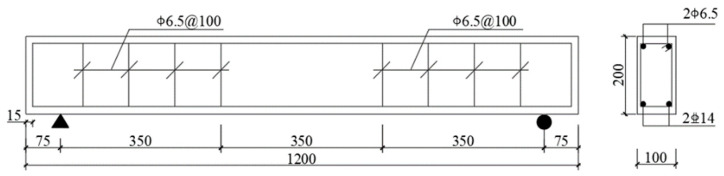
Size and reinforcement design of experimental beam.

For beam-scale validation, reinforced UHPC beams with the U1 matrix and 3.0% steel fibers of aspect ratio 65 were tested under four-point bending. The measured compressive and tensile strengths were 127.6 MPa and 7.7 MPa, respectively, and the main reinforcement and geometric details are shown in Figure 2. The beam-test arrangement followed configurations commonly reported in the literature.

#### 2.1.3. Preparation of Specimens and Components

Specimens and beams were mixed, cast, and cured following common UHPC preparation procedures. Dry materials were premixed, water and superplasticizer were added, and steel fibers were introduced gradually to avoid clustering. After casting and vibration, specimens were covered, demolded after 24 h, and cured for 28 days at 20 ± 2 °C and 95% relative humidity [33,34].

### 2.2. Test Method

#### 2.2.1. Uniaxial Tensile Test

**Figure 3:**
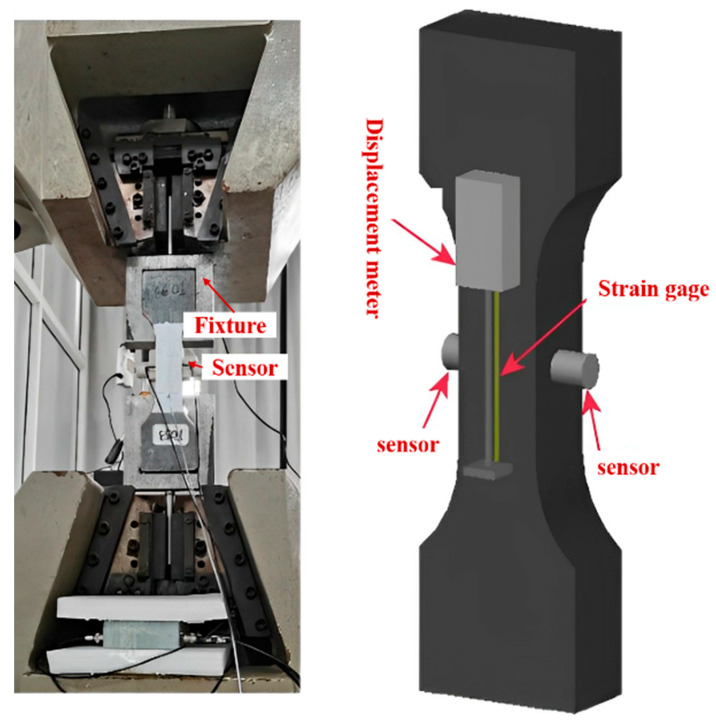
Sensor layout diagram.

Uniaxial tensile tests were conducted on a WAW-Y1000C (Wuhan Test Instrument Co., Ltd., Wuhan, China) electro-hydraulic servo machine using the fixture and gauge arrangement shown in Figure 3. A displacement rate of 0.1 mm/min was used, and valid results required crack localization within the 100 mm gauge region and a difference below 15% between displacement-gauge and strain-gauge readings in the elastic stage. These procedures are consistent with established UHPC direct-tension testing methods.

#### 2.2.2. AE Monitoring Test

AE monitoring was performed with sensors mounted on both sides of the gauge region, using a PCI-2 system and SR150N sensors (Figure 3). The acquisition setup, pencil-lead break check, 45 dB threshold, and 1 MHz sampling frequency were selected following ASTM E976-99 [35] and established AE practice. Cumulative AE count was used as the damage indicator because it provides a monotonic measure of progressive cracking.

#### 2.2.3. R-UHPC Beam Bending Test

**Figure 4:**
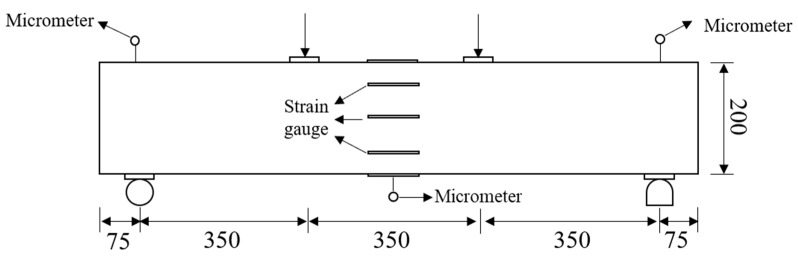
R-UHPC beam monitoring device layout.

R-UHPC beams were loaded under four-point bending with force control before yielding and displacement control, thereafter following established beam-test procedures. A 5 kN preload was applied, and load, deflection, and strain were recorded until compression-zone crushing, as illustrated in Figure 4.

### 2.3. Theoretical Framework and Model Derivation

#### 2.3.1. Acoustic-Emission-Based Damage Variable

AE activity is closely associated with the initiation and propagation of internal defects in concrete. During tensile loading, microcracking and interfacial damage release transient elastic waves, and the recorded AE parameters can therefore be used to characterize the damage development of UHPC. From the viewpoint of damage mechanics, material degradation can be described by the reduction in the effective load-bearing area caused by the accumulation of microdefects.

**Figure 5:**
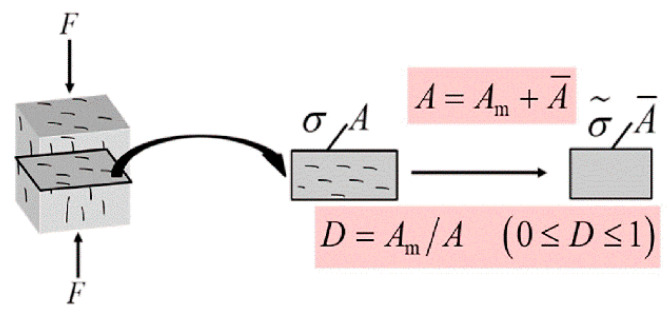
Schematic definition of the AE-based damage variable.

As illustrated in Figure 5, A represents the original cross-sectional area of the undamaged material, *Ā* denotes the remaining effective load-bearing area after damage, and *A_m_* = *A* − *Ā* is the damaged area. Thus, the damage variable *D* can be defined as the ratio of the damaged area to the original cross-sectional area. When *D* approaches 1, the material is assumed to have lost its effective load-bearing capacity.

(1) $$D=\frac{A_{m}}{A}$$

When an incremental damaged area *dA_m_* is generated in the cross-section, the corresponding increment of the damage variable can be expressed as follows:

(2) $$dD=\frac{dA_{m}}{dA}$$

Assuming that the AE count generated per unit damaged area of a microelement is n, the cumulative AE count associated with the damaged area can be related to the development of material deterioration. For the whole cross-section A, the total cumulative AE count corresponding to complete damage is denoted as Ntotal. Therefore, the relationship between the AE count increment and the damaged area can be written as:

(3) $$dN=\frac{N_{total}}{A}dA_{m}$$

It should be noted that *N*/*N_total_* represents a normalized indicator of relative damage evolution rather than a direct measurement of the actual cracked area. This definition relies on the assumption that, under the same sensor arrangement and acquisition threshold, cumulative AE counts are positively related to microcracking activity and the progressive loss of effective load-bearing area. The validity of this simplified damage indicator is further examined through Weibull fitting of the damage–strain curves and validation of the tensile stress–strain model. In Equation (4), N denotes the cumulative AE count at a specific loading time.

(4) $$D=\frac{N}{N_{total}}$$

where *N* is the cumulative AE counts at a specific time.

#### 2.3.2. Weibull Damage Evolution Function

A Weibull cumulative function was used to describe the relationship between tensile strain and the AE-based damage variable. Tensile failure of UHPC is a progressive process involving the activation of randomly distributed defects, such as matrix flaws, fiber–matrix interfacial defects, and microcracks. These defects do not fail simultaneously. Instead, relatively weak microelements are activated first, followed by the gradual development and coalescence of additional defects as the tensile strain increases. Therefore, the Weibull cumulative distribution is suitable for describing the probability-based evolution of tensile damage and provides a bounded damage variable ranging from 0 to 1. Although steel fibers can delay crack localization through bridging and pullout mechanisms, the cumulative AE count ratio still reflects the accumulation of damage-related acoustic events. For this reason, the Weibull function was adopted as a phenomenological damage evolution law and was verified by fitting the AE-derived damage–strain curves.

The cumulative AE counts recorded during the tensile failure process were processed to calculate the corresponding damage factor *D* according to Equation (5).

(5) $$D(\varepsilon )=1-\exp(-{\left(\varepsilon /m\right)}^{n})$$

#### 2.3.3. Tensile Damage Constitutive Model

According to Lemaitre’s strain equivalence hypothesis [36], the strain caused by nominal stress in a damaged material is assumed to be equivalent to the strain caused by effective stress in the corresponding undamaged material. This assumption provides the basis for relating the nominal stress, effective stress, elastic modulus, and damage variable. Under the tensile stress state considered in this study, the relationship can be expressed as Equation (6).

(6) σ=Eε(1−D)

By substituting the damage evolution function into the strain-equivalence relationship, the tensile damage constitutive equation of UHPC can be obtained as Equation (7).

(7) $$\sigma (\varepsilon )=E\varepsilon \exp(-{\left(\varepsilon /m\right)}^{n})$$

According to Equation (7), the tensile damage constitutive model is governed mainly by the distribution parameters m and n. Therefore, the influence of steel-fiber volume fraction and aspect ratio on the uniaxial tensile constitutive response of UHPC can be introduced indirectly by analyzing their effects on the damage evolution parameter.

To obtain a normalized expression of the constitutive relationship, Equation (7) was transformed into a dimensionless form by defining *y* = *σ*(*ε*)/*σ_p_* and *x* = *ε*/*ε_p_*. Based on the boundary conditions of the uniaxial tensile constitutive curve, Equation (8) was obtained, where *e* is the base of the natural logarithm.

(8) $$y=\sqrt[{n}]{e}x\exp(-\frac{x^{n}}{n});\;n=1/\ln(\frac{\varepsilon_{p}\sigma_{c}}{\varepsilon_{c}\sigma_{p}})$$

#### 2.3.4. Incorporation of Steel-Fiber Factor

The uniaxial tensile test data of UHPC with different steel fiber parameters were obtained in the test. The parameter *n* was obtained through the peak stress, peak strain, and initial elastic modulus according to Equation (8). In this study, the effects of steel fiber volume fraction ($V_{f}$) and the aspect ratio of steel fiber ($l_{f}/d_{f}$) on the plastic damage constitutive relation of UHPC were considered. The fiber factor *λ* [37,38] was introduced to reduce the variable, and the fiber factor was expressed as Equation (9).

(9) $$\lambda =V_{f}(l_{f}/d_{f})$$

## 3. Analysis of Damage Evolution Law of UHPC Under Uniaxial Tension at the Microscale

### 3.1. Characteristics of the AE Signal

**Figure 6:**
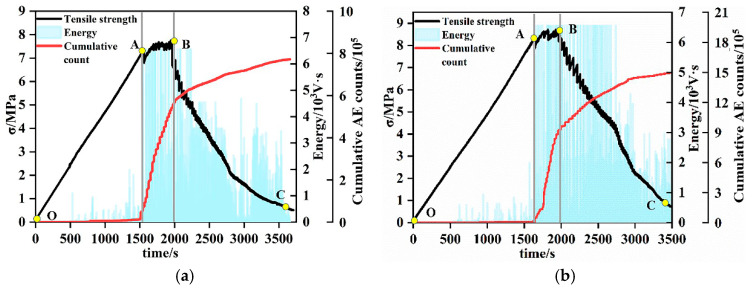
Evolution of tensile stress, AE cumulative counts, and AE energy during uniaxial tensile loading: (**a**) U1-65-1.0; (**b**) U1-65-1.5.

For specimen U1-65-0.5, the mechanical tensile response was considered valid, whereas the corresponding AE record was not used in the subsequent analysis. This is because discontinuous signal acquisition occurred in the AE channel during loading, which was probably caused by unstable sensor coupling or a temporary disturbance in signal transmission. Since the dog-bone specimens had already been destructively tested and the original mixing and curing conditions could not be reproduced exactly during revision, an additional AE test under identical conditions was not performed. As a result, the AE-based damage evolution analysis was conducted only for specimens U1-65-1.0 and U1-65-1.5. This limitation reduces the representativeness of AE verification at the lowest fiber volume fraction, but it does not affect the mechanical tensile analysis or the fiber-parameter calibration based on the complete tensile test series. The cumulative AE count and AE energy responses of U1-65-1.0 and U1-65-1.5 are presented in Figure 6. This section focuses on the evolution characteristics of cumulative AE count and AE energy during the entire uniaxial tensile loading process of UHPC.

As shown in Figure 6, the cumulative AE count and AE energy evolved consistently with the tensile stress response of specimens U1-65-1.0 and U1-65-1.5. Based on the stress development and AE response, the tensile process can be divided into three stages.

According to the test process, the change in stress can be divided into three stages:

(a) Elastic stage (path OA). In this stage, the tensile stress increases almost linearly, and the specimen mainly remains in the elastic deformation stage. Only a small number of AE events are detected, and the cumulative AE count remains at a low level. This indicates that limited microcracking occurs before the formation of the initial crack.
(b) Stable crack-propagation stage (path AB). After point A, matrix cracking begins and microcracks are progressively generated. This process is accompanied by a rapid increase in cumulative AE count and a relatively high level of AE energy. The damage in this stage is mainly associated with matrix cracking and the continuous formation of new microcracks.
(c) Unstable crack-propagation stage (path BC). In this stage, the growth rate of the cumulative AE count gradually decreases and tends to stabilize. Few new microcracks are formed, and damage development is mainly governed by the widening and propagation of dominant macroscopic cracks. The AE response in this stage is primarily related to fiber pullout and fiber–matrix interaction [39].

### 3.2. AE-Based Damage Evolution Analysis

**Figure 7:**
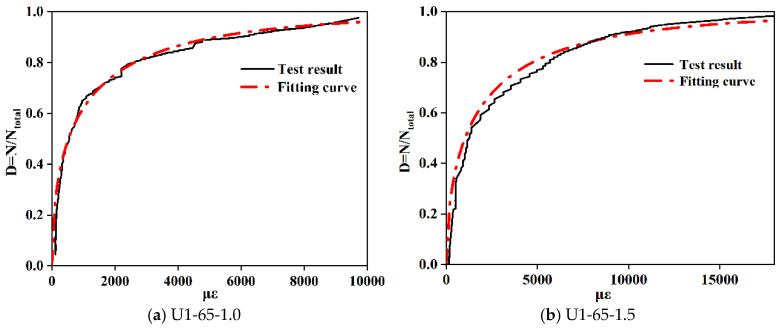
Damage-strain relation curve.

Using the AE-based damage variable defined in Section 2.3.1, the damage–strain curves of specimens U1-65-1.0 and U1-65-1.5 were obtained. As shown in Figure 7, the experimental damage evolution can be effectively described by the Weibull cumulative function introduced in Section 2.3.2, indicating that the proposed function is suitable for characterizing the progressive tensile damage evolution of UHPC under the tested conditions.

## 4. Analysis of the Influence of Mesoscale Steel Fiber Parameters on the Constitutive Relation of UHPC Under Uniaxial Tension

### 4.1. Effect of Steel Fiber on Crack Initiation and Failure Mode

**Figure 8:**
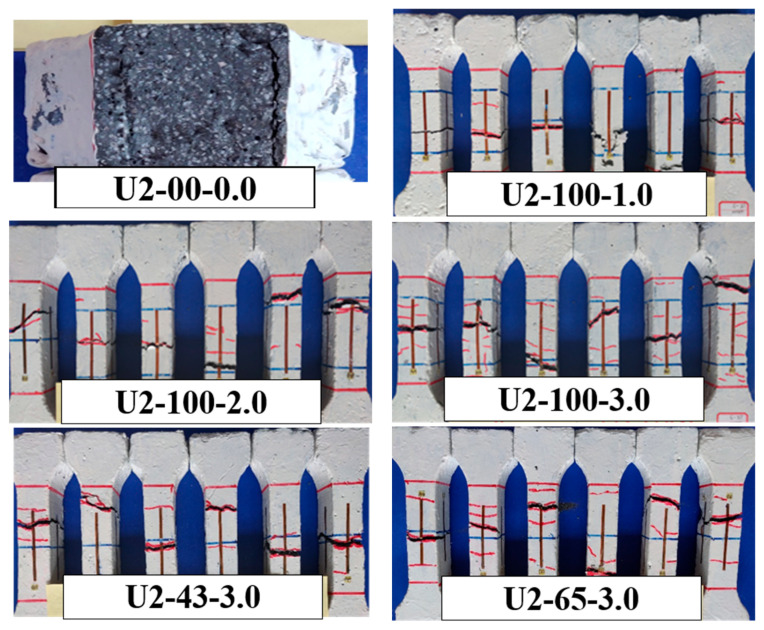
Failure mode diagram of UHPC under different volume fractions.

The typical failure patterns of UHPC specimens under uniaxial tensile testing (UTT) are presented in Figure 8. As the steel-fiber volume fraction increases, the failure behavior gradually changes from brittle fracture to more ductile failure. In general, specimens with a higher fiber volume fraction and a larger fiber aspect ratio exhibit more microcracks in the failure region, indicating a more pronounced ductile response. After the first crack appears, the bridging action between the steel fibers and the matrix restrains further crack opening and propagation [40,41,42]. This bridging effect allows cracks to develop more steadily and promotes the formation of additional cracks in secondary weak sections. With increasing fiber content, the crack-bridging capacity becomes stronger, resulting in more distributed cracking and a failure mode closer to ductile failure. Under continued loading, the dominant crack gradually widens, the tensile stress reaches its peak, and the fibers are progressively pulled out until the specimen finally fails.

### 4.2. Effect of Steel Fiber on a Stress–Strain Curve Under Uniaxial Tension

**Figure 9:**
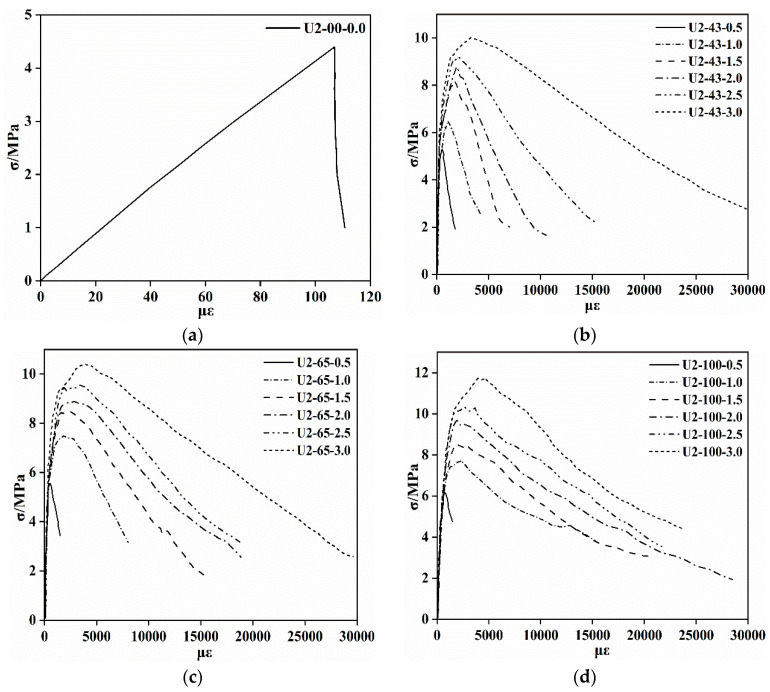
Uniaxial tensile stress–strain curves of UHPC with different steel-fiber aspect ratios and volume fractions: (**a**) plain matrix; (**b**) aspect ratio 43; (**c**) aspect ratio 65; (**d**) aspect ratio 100.

Figure 9 presents the uniaxial tensile stress–strain curves of UHPC with different steel-fiber aspect ratios and volume fractions. The results indicate that steel fibers have a significant influence on the tensile stress–strain response of UHPC. At a relatively low fiber volume fraction, the specimens mainly exhibit stress-softening behavior after cracking. When the fiber volume fraction exceeds a critical level, the tensile response changes to a pseudo-strain-hardening behavior. The tensile strength increases with increasing steel-fiber volume fraction. The peak tensile strain increases markedly near the critical fiber volume fraction and then continues to increase approximately linearly as the fiber volume fraction further increases.

It should be noted that this study considered steel-fiber volume fraction and aspect ratio, but fiber orientation and spatial dispersion were not directly quantified. Since fiber orientation and dispersion strongly affect crack-bridging efficiency and tensile strain-hardening behavior, the fiber factor adopted in this study should be regarded as an engineering simplification. Future work should combine image analysis, X-ray computed tomography, or section-based fiber counting with AE monitoring to further improve the description of fiber effects in the constitutive model. Nevertheless, the present study extends existing tensile constitutive modeling by introducing a fiber factor into the tensile damage constitutive equation and linking it with AE-based damage evolution and beam-scale finite element validation.

## 5. Model Calibration, Validation, and Beam-Scale Application

### 5.1. Calibration of the Fiber-Dependent Constitutive Parameter

According to Equation (8), the plastic damage constitutive relationship is mainly controlled by the model parameter n. In this study, n was back-calculated from the measured peak stress, peak strain, and initial elastic modulus of each tensile curve. The corresponding fiber factor *λ* was determined from the steel-fiber volume fraction and aspect ratio. The results show that n decreases as *λ* increases, indicating that steel-fiber parameters have a clear influence on the tensile damage constitutive response. Therefore, nonlinear least-squares regression was performed in Origin to establish the relationship between *n* and *λ*. As expressed in Equation (10), the initial regression gave R^2^ = 0.82, suggesting a clear correlation between the two parameters, although some scatter remained. This scatter may be related to fiber orientation, fiber dispersion, matrix variability, and experimental uncertainty. To reduce the risk of overfitting to the present test results, the regression equation was further corrected using both the experimental data obtained in this study and 14 independent datasets collected from the literature.

(10) $$n=0.36+0.62e^{-2.28\lambda },0<\lambda <3$$

### 5.2. Verification and Correction of Multiscale Constitutive Equations

**Figure 10:**
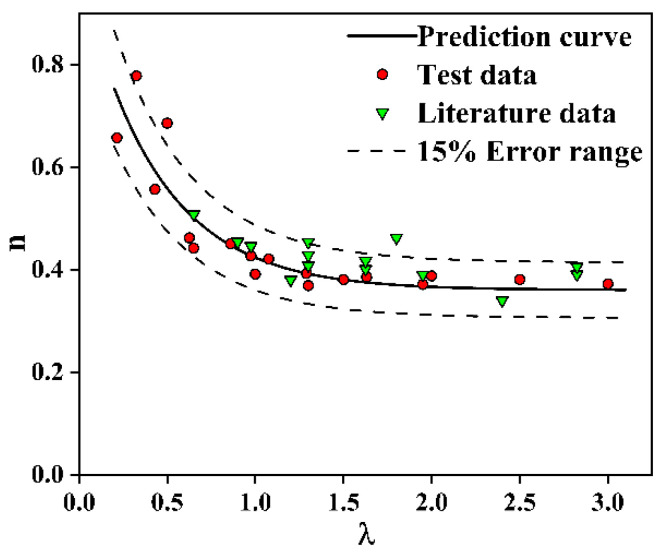
*n*-value regression analysis.

To verify the applicability of the proposed prediction method for coefficient n, 14 uniaxial tensile datasets reported in the literature were collected and analyzed. These datasets cover different UHPC matrix systems, steel-fiber volume fractions, and fiber aspect ratios [3,4,7,43,44,45,46]. According to Equation (8), the predicted n values were compared with those back-calculated from the verification test data. As shown in Figure 10, the initial prediction equation produced a mean relative error of 3.9% and a standard deviation of 5.1%, with all data points falling within the ±15% error range. These results indicate that Equation (10) has acceptable adaptability to different matrix conditions and steel-fiber parameters.

The collected literature data and the experimental results obtained in this study were jointly used for regression analysis to improve the accuracy of the prediction equation. The corrected relationship is expressed as Equation (11).

(11) $$n=0.38+0.60e^{-2.37\lambda },0<\lambda <3$$

Therefore, the plastic damage constitutive relation can be expressed as Equation (12).

(12) $$\begin{array}{c} y=\sqrt[{n}]{e}x\exp(-\frac{x^{n}}{n});\\ n=0.38+0.60e^{-2.37\lambda },0<\lambda <3 \end{array}$$

**Figure 11:**
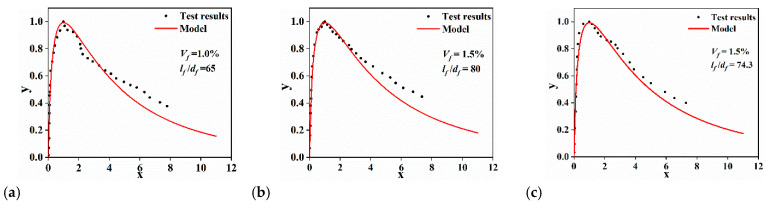

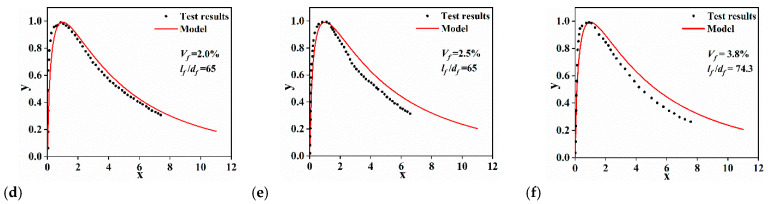
Comparison between experimental and predicted tensile stress–strain curves: (**a**) Augusto et al. [10]; (**b**,**c**) Le et al. [7]; (**d**,**e**) Wille et al. [44]; (**f**) Shen et al. [4].

After correction, the mean relative error of the estimated coefficient n decreased to 2.6%, and the standard deviation decreased to 4.1%, indicating that the prediction accuracy was further improved. To verify whether Equation (12) can effectively predict the uniaxial tensile constitutive curve of UHPC, the model predictions were compared with the experimental results collected from the literature, as shown in Figure 11. The validation database included 14 independent uniaxial tensile datasets reported in previous studies, covering different UHPC matrix systems, steel-fiber volume fractions, and fiber aspect ratios [3,4,7,43,44,45,46].

The model accuracy was quantitatively evaluated using the relative error, mean relative error, standard deviation of the relative error, and coefficient of determination. For the corrected prediction equation, the mean relative error was 2.6%, the standard deviation was 4.1%, and the fitted tensile stress–strain curves generally showed R^2^ values greater than 0.85. The predicted curves were close to the measured results, indicating that the constitutive model established by Equation (12) can reasonably describe the uniaxial tensile response of UHPC. In addition, the model showed good fitting performance for UHPC mixtures with different matrix compositions, steel-fiber aspect ratios, and steel-fiber volume fractions. Since the number of valid tensile curves for some mixtures was limited, error bars were not added to all stress–strain curves. Additional repeated tensile tests are recommended in future work to further quantify specimen-to-specimen variability.

### 5.3. Comparison with Existing Tensile Constitutive Models

Compared with existing tensile constitutive models that are mainly calibrated from macroscopic stress–strain curves [8,9,10,11,12,13], the proposed model introduces both an AE-based damage variable and a steel-fiber factor, thereby providing a clearer physical connection among damage evolution, fiber reinforcement, and tensile constitutive response. Piecewise empirical models can reproduce specific experimental curves, but their coefficients usually need to be recalibrated for different mixtures. Damage models based on stiffness degradation or secant modulus reduction can describe macroscopic mechanical deterioration, but they do not directly incorporate real-time damage information obtained during loading. In contrast, the present model defines the damage variable using the AE cumulative count ratio and relates the constitutive parameter to steel-fiber volume fraction and aspect ratio. However, the effects of fiber orientation, fiber dispersion, and AE threshold sensitivity are still simplified in the present model, and these limitations should be further quantified in future studies.

### 5.4. Beam-Scale Finite Element Validation

To evaluate the applicability of the proposed UHPC tensile constitutive model at the member scale, a three-dimensional finite element model of the reinforced UHPC beam was established in ABAQUS (2022, Dassault Systèmes, Vélizy-Villacoublay, France) The failure characteristics of the R-UHPC beam and the load–midspan deflection response were selected as the main evaluation indices. The numerical results were then compared with the experimental observations to assess the effectiveness of the proposed constitutive model in structural analysis.

**Figure 12:**
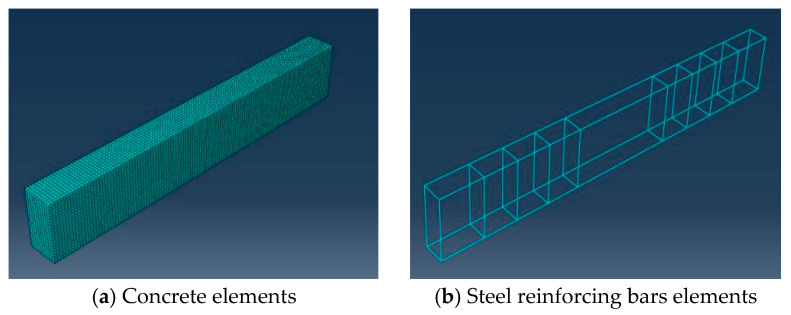
Meshed beam specimen.

In the finite element model, the beam geometry was defined according to the actual dimensions of the test beam, and the mesh size was set to 10 mm. UHPC was simulated using three-dimensional eight-node linear solid elements, whereas the longitudinal steel bars and stirrups were modeled using two-node linear three-dimensional truss elements, as shown in Figure 12. The material properties were assigned based on the measured experimental values. The elastic modulus of the steel plates at the loading and support positions was taken as 210 GPa. The reinforcement cage was embedded in the UHPC beam as embedded elements, and the loading and support plates were tied to the beam surface. The left support was modeled as a fixed hinge support, while the right support was modeled as a vertical roller support.

The nonlinear behavior of UHPC was simulated using the concrete damaged plasticity model (CDPM) in ABAQUS. The main material inputs included the elastic modulus, Poisson’s ratio, compressive strength, tensile strength, tensile stress–strain relationship, compressive stress–strain relationship, and CDPM plasticity parameters. The tensile behavior of UHPC was defined according to the damage constitutive relationship proposed in Equation (12), whereas the compressive behavior was determined based on the experimental compressive test data. The compressive input and CDPM plasticity parameters were assigned according to the measured compressive strength and relevant recommendations in the literature, as summarized in Table 4.

The beam, loading plates, and supports were modeled according to the experimental geometry. The four-point bending boundary condition was reproduced by constraining the vertical displacement at the supports and applying monotonic loading through the loading plates. The nonlinear analysis in ABAQUS was conducted using automatic incrementation, and the convergence behavior was checked during the calculation to ensure stable load–deflection results.

**Figure 13:**
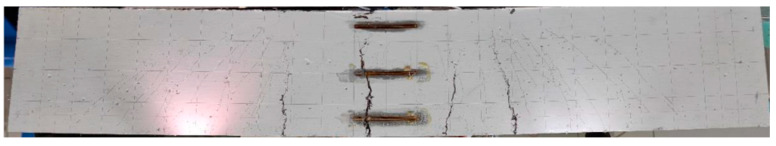

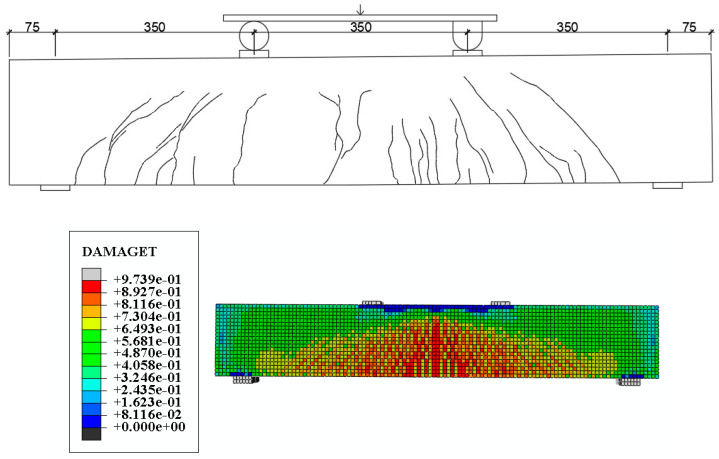
Damage pattern of reinforced UHPC beams.

The crack diagram of the test beam and its finite element model, as shown in Figure 13. The results show that in each failure stage of the R-UHPC beam, the shape and development characteristics of cracks are in good agreement with the damage conditions of the elements in the finite element model.

**Figure 14:**
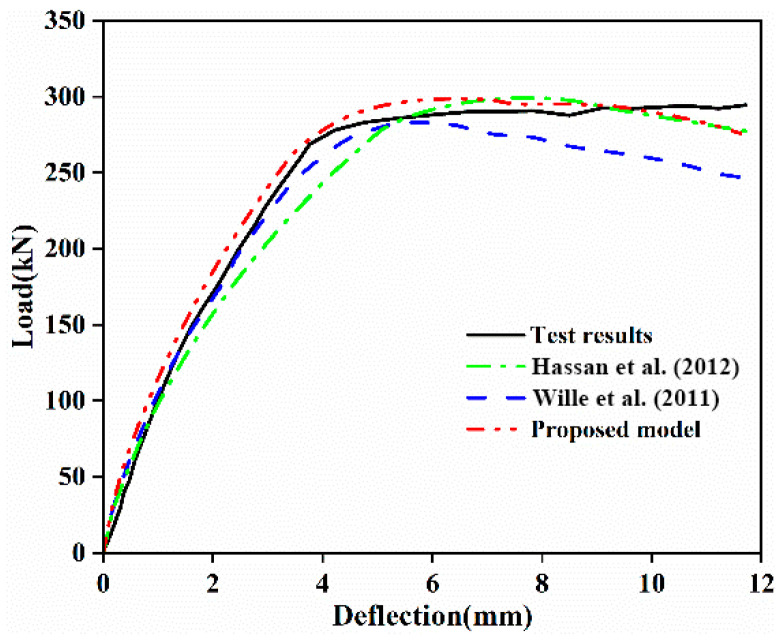
Load-deflection curve for the reinforced UHPC beam [44,47].

To further verify the accuracy of the proposed damage constitutive relationship, tensile constitutive models reported in the literature [44,47] were also collected for comparison. The load–deflection curve of the R-UHPC beam obtained from the experiment was compared with the finite element simulation results, as shown in Figure 14. The comparison indicates that the simulation based on the damage constitutive model proposed in this study shows the closest agreement with the experimental beam response.

The tensile constitutive relationship proposed in this study was calibrated using dog-bone specimens with a tensile section of 50 mm × 50 mm. When this relationship was directly applied to the reinforced UHPC beam with a section of 100 mm × 200 mm, the simulated load–deflection response was higher than the experimental result. This discrepancy may be associated with the transition from specimen-scale direct tension to member-scale bending, where nonuniform stress distribution, crack localization, reinforcement interaction, and possible differences in fiber orientation may affect the tensile response. Therefore, a member-scale reduction coefficient μ was introduced to reduce the tensile stress branch of the constitutive model used in the beam simulation. By comparing the simulated and measured load–deflection curves, μ = 0.84 was adopted in this study. This coefficient should be regarded as an empirical calibration factor for the tested beam geometry, reinforcement layout, material properties, and loading condition, rather than a universal size-effect coefficient. With this correction, the UHPC uniaxial tensile constitutive model considering steel-fiber parameters becomes more suitable for describing the tensile mechanical behavior of R-UHPC beams and can provide a useful reference for the structural analysis and design of UHPC members.

(13) $$\begin{array}{c} y=\mu \;\sqrt[{n}]{e}x\exp(-\frac{x^{n}}{n});\\ n=0.38+0.60e^{-2.37\lambda },0<\lambda <3 \end{array}$$

## 6. Conclusions

(1) The AE cumulative count ratio can be used as a damage variable to characterize the tensile damage evolution of UHPC under uniaxial tension. The damage–strain relationship obtained from AE monitoring was well described by a Weibull cumulative distribution. The proposed model quantified the influence of steel-fiber volume fraction and aspect ratio through a fiber factor, allowing the tensile constitutive parameter to be predicted without relying solely on curve-by-curve empirical fitting. After correction using both the present experimental data and literature data, the model achieved a mean relative error of 2.6% and a standard deviation of 4.1% for the predicted model parameter.
(2) The proposed model quantified the effects of steel-fiber volume fraction and aspect ratio through a fiber factor, enabling the tensile constitutive parameter to be predicted without relying solely on curve-by-curve empirical fitting. After correction using both the present experimental data and literature data, the model achieved a mean relative error of 2.6% and a standard deviation of 4.1% for the predicted model parameter.
(3) Beam-scale finite element analysis showed that introducing a reduction coefficient of μ = 0.84 improved the agreement between the simulated and measured load–deflection responses. This coefficient should be regarded as an empirical member-scale calibration factor for the beam geometry, reinforcement layout, material properties, and loading condition considered in this study.
(4) The proposed model establishes a connection among AE-based damage evolution, steel-fiber parameters, and member-scale finite element simulation. However, its application is still limited by the absence of direct fiber-orientation characterization, AE threshold-sensitivity analysis, and broader beam-scale validation. These issues should be further investigated in future studies.

## Figures and Tables

**Table 1:** Mix proportions of the U1 matrix by mass ratio.

Cement		Silica Fume	Sand	Superplasticizer	Water
P.O52.5	P.C42.5				
0.700	0.300	0.180	1.180	0.024	0.188

**Table 2:** Mix proportions of the U2 matrix by mass ratio.

Cement (P.O42.5)	Silica Fume	Sand	Superplasticizer	Water
1.0	0.3	1.17	0.025	0.234

**Table 3:** Experimental matrix and specimen labels.

Series	Matrix	Fiber Aspect Ratio	Fiber Volume Fraction/%	Specimen Labels	Purpose
U1-65	U1	65	0.5, 1.0, 1.5	U1-65-0.5 to U1-65-1.5	AE damage analysis
U2-43	U2	43	0.5, 1.0, 1.5, 2.0, 2.5, 3.0	U2-43-0.5 to U2-43-3.0	Fiber-parameter analysis
U2-65	U2	65	0.5, 1.0, 1.5, 2.0, 2.5, 3.0	U2-65-0.5 to U2-65-3.0	Fiber-parameter analysis
U2-100	U2	100	0.5, 1.0, 1.5, 2.0, 2.5, 3.0	U2-100-0.5 to U2-100-3.0	Fiber-parameter analysis
Beam	U1	65	3.0	R-UHPC beam	Beam-scale validation

**Table 4:** UHPC material input parameters used in ABAQUS CDPM.

Dilatation Angle	Eccentricity	$\sigma_{b0}$/$\sigma_{c0}$	K	Viscosity Parameter
36	0.1	1.16	0.667	0

## Data Availability

The original contributions presented in this study are included in the article. Further inquiries can be directed to the corresponding author.

## Abbreviations

UHPC, ultra-high-performance concrete; UHPFRC, ultra-high-performance fiber-reinforced concrete; AE, acoustic emission; UTT, uniaxial tensile test; R-UHPC, reinforced ultra-high-performance concrete; CDPM, concrete damaged plasticity model; FEM, finite element method; XRD, X-ray diffraction; XRF, X-ray fluorescence; PSD, particle-size distribution; SP, superplasticizer; SFiber, steel fiber; SiF, silica fume.
